# First Neonates with Vertical Transmission of SARS-CoV-2 Infection in Late Pregnancy in West Part of Romania: Case Series

**DOI:** 10.3390/diagnostics12071668

**Published:** 2022-07-09

**Authors:** Daniela Iacob, Ileana Enatescu, Mirabela Dima, Elena Bernad, Manuela Pantea, Daiana Bozgan, Sandor Bernad, Marius Craina

**Affiliations:** 1 Department of Neonatology, “Victor Babes” University of Medicine and Pharmacy, EftimieMurgu Square no 2, 300041 Timisoara, Romania; iacob.daniela@umft.ro (D.I.); enatescu.ileana@umft.ro (I.E.); dima_mirabela@yahoo.com (M.D.); manu.pantea@gmail.com (M.P.); 2 Department of Obstetrics and Gynecology, “Victor Babes” University of Medicine and Pharmacy, EftimieMurgu Square no 2, 300041 Timisoara, Romania; craina.marius@umft.ro; 3 Clinic of Neonatology, “PiusBrinzeu” County Emergency Hospital, 300723 Timisoara, Romania; daiana.bozgan@yahoo.com; 4 Romanian Academy Timisoara Branch, Mihai Viteazul Avenue, 24, 300275 Timisoara, Romania; sandor.bernad@upt.ro

**Keywords:** neonates, SARS-CoV-2 infection, COVID-19, vertical transmission, late pregnancy

## Abstract

The Coronavirus disease 2019 (COVID-19) pandemic has exposed the vulnerable neonatal population to unknown risks. Given that herd immunity is has not been reached, the entire population is susceptible to Severe Acute Respiratory Syndrome Coronavirus 2 Virus(SARS-CoV-2) infection. The arising concern about the vertical transmission of neonatal complications caused by the novel coronavirus is a continuous challenge for managing newborns, considering the rare cases and unclear guidelines. Therefore, a retrospective study was conducted in a tertiary unit from Timisoara, Romania. Of the 283 newborns born during the study period, only 3 neonates were diagnosed with SARS-CoV-2 infection in the first 24 h of life (DOL-0). The present study plans to identify the findings, including clinical features, laboratory characteristics, and outcomes of newborns with vertical transmission of SARS-CoV-2. All infected neonates were confirmed with COVID-19 by Reverse-Transcriptase Polymerase Chain Reaction (RT-PCR) from nasal aspirates and were isolated in the neonatology department. They were the first and the only neonate infected at birth from the West part of Romania. The clinical findings were unremarkable except for one neonate who developed mild respiratory distress syndrome. Elevated IgG-specific anti-SARS-CoV-2 serum levels were found in one newborn. Swab samples in DOL-0 strengthened the awareness of vertical transmission, although peripartum SARS-CoV-2 infection does not seem responsible for severe symptoms. We conclude that vertical transmission is rare in late pregnancy. Even if the studied newborns showed mild forms of COVID-19, it is essential to note that newborns represent a particular category of patients. More studies are needed to complete the observations of this study.

## 1. Introduction

Coronavirus disease 2019 (COVID-19), caused by the Severe Acute Respiratory Syndrome Coronavirus 2 Virus (SARS-CoV-2), was initially discovered in China in December 2019. With the continued rise of COVID-19 cases the World Health Organization (WHO) declared this a pandemic on 11 March 2020 [1]. The first case of SARS-CoV-2 infection in Romania was declared on 26 February 2020. Despite the installed measures and the national vaccination program implemented in late 2020, infections have been constantly increasing. On 2 January 2022, more than 2.8 million infections of SARS-CoV-2 and 65,428 deaths were reported in Romania [2]. The latest studies consider specific categories of people at increased risk of severe infection, among which are pregnant women [3], with side effects of maternal infection extending to the newborn through vertical transmission [4]. SARS-CoV-2 could be directly transmitted from mother to child during pregnancy [5,6,7,8]. Evidence on the vertical transmission of SARS-CoV-2 infection among newborns is an arising challenge in prevention and treatment strategies. WHO elaborated on a scientific brief in which explanations about the means of transmission from mother to fetus, whether it is in utero, intrapartum, or postnatal, are to be found. The classifications have not been updated due to the ongoing pandemic, and more data are bound to emerge [9].

Several studies show that the vertical transmission of SARS-CoV-2 is rare but possible, especially during the third trimester of pregnancy [8,10,11]. In these cases, the timing and mode of delivery should be determined by maternal disease status or other obstetrical problems [12,13].

Transplacental transmissions of the infection may lead to symptoms typical of SARS-CoV-2 infection or neurological signs, but most newborns develop mild forms of the disease or are asymptomatic [14].

Once the diagnosis of infection has been established, proper case management is crucial, which includes isolating the newborn with the mother if their health permits this, evaluating the newborn to determine the severity of the disease, performing imaging investigations if necessary, and applying appropriate therapeutic measures [15].

This paper presents three cases of newborns delivered to COVID-19-positive mothers whom were diagnosed by real-time Reverse-Transcriptase Polymerase Chain Reaction (RT-PCR) with SARS-CoV-2 infection. This study aims to evaluate neonatal clinical and paraclinical characteristics and their outcomes due to vertical transmission of SARS-CoV-2.

## 2. Materials and Methods

### 2.1. Objects and Data Collection

We present a case series that includes three neonates born from SARS-CoV-2 positive mothers with a positive RT-PCR for SARS-CoV-2 collected by an NP aspirate within 24 h of life—day zero(DOL-0 [HOL-0 to HOL-24]). They were delivered in the largest tertiary maternity units in Western Romania from the County Emergency Hospital “PiusBrinzeu” Timisoara (CEHPBT). The state government designated it as a COVID-19 support hospital in March 2020. Between 1 April 2020 and 31 December 2021, 283 neonates were born from mothers with COVID-19. We described the characteristics of the three neonates, including demographic information, data about the early neonatal period, and clinical and laboratory findings.

### 2.2. Procedures

A team of obstetricians, neonatologists, anesthesiologists, and nurses attended the births. The team used equipment as the protocol suggests since all pregnant women tested positive for SARS-CoV-2 infection. All healthcare providers who wore protective equipment were allowed to enter the COVID-19 neonatal unit during hospitalization. The Apgar score was used to assess the clinical status of the newborn. The neonatologist evaluated the newborns in the delivery room. Immediately after birth, nasopharyngeal (NP) swabs were collected from all the newborns in the delivery room. The newborns had been placed in a transition ward until the test results wereobtained. Until the result came, the newborns were placed in the ward for isolation. Clinical examination, vital function monitorization, and laboratory tests were performed.

### 2.3. Specimen Collection and Virus Detection

NP probes were collected from the newborns at birth and then rectal probes were collected after DOL-0. OP and NP swabs were collected at the admission of the pregnant women. After the infection, diagnostics were established.We also collected probes from the vagina and rectum of the pregnant women. The probes were taken into sterile tubes with viral transport media and sent to specialized units named in the Technical Norms for implementing national health programs. The National Center for Surveillance and Control of Communicable Diseases from Bucharest, Romania, elaborated strict recommendations related to collecting, transporting, and processing samples in the laboratory to prevent the spread of SARS-CoV-2 infection [16]. The samples were processed by RT-PCR. In these cases, no samples were taken from the placenta, amniotic liquid, or umbilical cord. Specific immunoglobulin G (IgG anti SpikeS1) and TOTAL Anti-SARS-CoV-2 antibody (IgA IgM IgG) were collected fromthe newborns. With respect tothe mothers, no COVID-19 antibodies were dosed.

### 2.4. Ethics Approval

Because the study involved newborns, each mother was informed about all aspects of the present study. They were informed and referredto all the aspects contained in the informed consent form regarding biological sampling, pregnancy monitoring, birth care, postpartum management, and SARS-CoV-2 infection management on the recommendation of the infectious disease specialist for both them and the newborns. Patients also agreed to participate in medical education and research. After being informed, the patients signed all agreements. The Ethics Committee of CEHPBT approved the study (272/14 October 2021).

## 3. Results

Materno-fetal infection occurred in 1.06% of neonates in the studied period if we refer to patients in the third trimester of pregnancy. The newborns were evaluated from birth until discharge. The demographic characteristics and anthropometric measurements are shown in Table 1. All were delivered at term and were considered appropriate for gestational age (AGA). The first two were born in our clinic from SARS-CoV-2 positive mothers admitted to our unit; Newborn 3 was transferred from a secondary maternity hospital after birth, as they were diagnosed with COVID-19. Newborn 1 was born by vaginal delivery. The two others were born by non-elective cesarean section based on medical indications, namely labor dystocia and fetal hypoxia. All newborns tolerated the birthing process well, as demonstrated by the Apgar score at 1 min: 8, 9, and 9. At 5 min, the Apgar score was 10 in all cases, emphasizing fetal wellbeing outside the mother’s womb.

All newborns were tested for RT-PCR at birth and the NP probe results reflected positive for two out of three cases. The first patient had an inconclusive result. This test was repeated as the hospital laboratory suggested within DOL-0, and the second time resulted in a positive.

Patient 3 had two positive RT-PCR NP swab tests because the first one was collected and performed at the place of birth—a secondary maternity unit. He was transferred within DOL-0, and we collected an NP swab test at admission into our Clinic of Neonatology. The national protocol according to testing for COVID-19 suggests that if the first RT-PCR test is positive, it should not be repeated since it is equivalent to the disease. An evaluation for SARS-CoV-2 infection of the newborn was performed with two swab tests—NP and rectal. All patients were tested for RT-PCR at birth and the NP results were positive for Newborns 2 and 3. The first patient had an inconclusive result, repeated as the hospital laboratory suggested within the first 24 h of life, and the second test came back positive. The detection of the virus by RT-PCR in rectal swab was made only for two patients and might not suggest vertical transmission because the samples were provided after DOL—zero. All mothers had positive test results for SARS-CoV-2 infection at admission before giving birth or immediately after birth.

All mothers were tested positive for SARS-CoV-2 infection at admission before giving birth. Two of them were asymptomatic while one had mild symptoms. After the evaluation in the delivery room, the newborns were admitted to the neonatal unit. A first NP swab test for detection of the SARS-CoV-2 virus was collected. Until the results were obtained, the newborns are separated from their mothers. They were placed in a servo-controlled incubator under continuous cardiorespiratory and temperature monitoring. None of them presented abnormal values for oxygen saturation, heart rate, and temperature. Patient one, delivered from the symptomatic mother, developed mild respiratory distress syndrome (RDS)—tachypnea (65 breaths/min), minimal intercostal retraction, and audible expiratory grunt—assessed with Silverman Score at three. Supplemental oxygen was needed for two days until complete respiratory improvement appeared. RDS limits enteral nutrition, and parenteral nutrition starts via the peripheral route. Broad-spectrum antibiotic therapy was provided intravenously for three days, with the patient evolution being favorable during hospitalization. The other two newborns were asymptomatic.

Neonatal clinical findings during the hospitalization period are summed up in Table 2, showing individualized treatment and care. For example, Patient 1 received two-day oxygen therapy, antibiotic therapy for three days, parenteral nutrition for two days, phototherapy with protective goggles, borax glycerin solution, and protective cream. Patients 2 and 3 received borax glycerin solutions and protective cream. Rooming-in and breastfeeding were considered adequate, with safety contact precautions, such as wearing a mask when in close contact with the neonates and performing proper hand hygiene while handling or breastfeeding.

The blood tests showed no abnormal values for the complete blood count. Elevated C-reactive protein (CRP) levels were found for patients one and two. Patient one’s clinical evaluation was linked to an increased CRP level. This determined the choice for antibiotic therapy. As a result, a decrease in CRP serum levels was observed. The therapeutic management approach for patient two was different. The neonatal team correlated with the clinical signs and serum levels of CRP, aspartate aminotransferase, and lactate dehydrogenase, which suggested perinatal hypoxia. Therefore, they decided to see the dynamics of CRP levels without using antibiotics. Two out of three patients had elevated ferritin levels. One of the newborns had specific immunoglobulin G positive levels that suggest in utero antibody transfer from the mother through the placenta(Table 3).

The evolution was favorable in all cases. According to the vaccine recommendations of the National Institute of Health in Romania, they received anti-hepatitis B and antituberculosis immunizations during the hospital stay [17].

**Figure 1:**
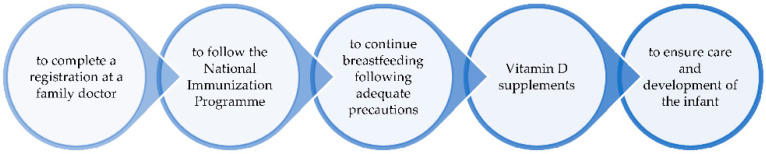
Recommendations at discharge.

After seven days of medical care, they were discharged in isolation at home for up to 14 days after the first positive RT PCR SARS-CoV-2 test. According to the current legislation from Romania regarding the measures applied in the field of public health in situations of epidemiological risk of infection with the SARS-CoV-2 virus, patients should not be retested after the isolation period is over. Moreover, patients are cured after 14 days in the absence of clinical manifestations [18]. Recommendations at discharge are shown in Figure 1.

## 4. Discussion

The maternal-to-fetal transmission of SARS-CoV-2 is described as rare. However, in a systematic review, Kotlyar et al. included 936 tested newborns born to pregnant women diagnosed with COVID-19 during the third trimester. The vertical transmission was estimated to be approximately 3.2% [8]. Our group’s results have shown that SARS-CoV-2 vertical infections in the third trimester of pregnancy are rare (1.06%).

Although there are many breakthrough studies regarding newborns diagnosed with COVID-19, our study shows three neonates with positive RT-PCR SARS-CoV-2 test delivered from COVID-19 diagnosed mothers. The rate of neonatal infection, neonatal deaths, and maternal deaths is no more significant when the mother gives birth through vaginal delivery. However, it is not known whether vaginal delivery increases the risk of mother-to-child intrapartum transmission and whether uterine contraction could increase the possibility of transplacental transmission of infection. A review by Cai et al. concludes that there are insufficient data to support the fact that, in the case of pregnant women with confirmed COVID-19 infection, a cesarean delivery would be more appropriate than vaginal delivery in preventing a possible vertical transmission. Therefore, it is recommended that the mode of birth takes into account the severity of the infection and obstetric indications [19].

A review by Trippella (2020) presents nine newborns with positive RT-PCR tests, seven isolated from the mother and fed with milk formula, and two in rooming-in and breastfed.However, the tests were collected in the first 36–48 h of life and excluding a nosocomial infection was impossible [20]. In our case, the NP swab tests were collected at birth to rule out a potential nosocomial infection. In addition, the transmission of the virus through droplets can also be excluded, given that the medical staff wore personal protective equipment. Our testing method—the first swab test collected in DOL-0—is similar to other studies in the literature [5,21].

A COVID-19-designated hospital in Italy had the same approach to testing infants born to SARS-CoV-2 infected mothers. Both NP and rectal swab tests were performed at admission and repeated until two consecutive negative tests [22]. In this study, the authors described the persistence of the coronaviruses in the gastrointestinal tract after birth. This type of double RT-PCR testing was also used in the research letter of Zeng, which presents a batch of 33 neonates born to mothers with SARS-CoV-2 infection, of which three were identified with COVID-19 disease based on RT-PCR SARS-CoV-2 NP and rectal tests [23]. The combined NP and anal swab testing of the newborns in our clinic showed us that both results were positive in the first described case. It is to be specified that patient 2 tested positive with the NP swab and tested negative with the rectal test. The anal swab was unavailable at Newborn 3. In our group, detecting the virus by RT-PCR in rectal swabs only for two patients might not suggest vertical transmission because the samples were provided after DOL—zero.

The case report published by Dong describes a neonate born to COVID-19 confirmed mother, who had negative serial RT-PCR NP swab tests but elevated levels of IgG and IgM SARS-CoV-2 [24]. However, the possibility of vertical transmission of the novel coronavirus from the mother to the fetus should not be neglected. Kotlyar performed a systematic review and meta-analysis on the vertical transmission of COVID-19, showing evidence of transmission when the maternal infection appears in the third trimester of pregnancy [8]. In addition, studies report the possibility of transplacental transfer of immunoglobulins, especially IgG-type and IgM to a lesser extent [25,26].

To better understand the possible transmission pathway, we have quantitatively collected IgG-specific antibodies—anti SpikeS1. No elevated antibodies were found in patients 1 and 2 blood samples, but patient 3 showed a value about 90 times higher than the standard value of IgG anti-Spike S1. Unfortunately, during the three newborns’ hospitalization period, it was impossible to determine the serum level of IgM SARS-CoV-2 in our hospital.

The transmission of SARS-CoV-2 by exposure to respiratory fluids is now well known [27]. Therefore, it remains to be seen whether the benefits of rooming-in outweigh the risk of horizontal transmission between mother and newborn. The differences in the postnatal care guidelines have led to the development of national and local management protocols. The Union of European Neonatal and Perinatal Societies (UENPS) and WHO guidelines recommend rooming-in and breastfeeding following adequate hand hygiene and breastfeeding precautions. On the other hand, the American Academy of Pediatrics (AAP) recommends separation between the mother and the infant but encourages feeding of expressed breast milk. The Chinese guidelines highlight the separation of the newborn from the mother and no maternal breast milk use [28]. Newborns born to mothers with confirmed SARS-CoV-2 infection should be considered patients under investigation until test results arrive [29]. As the pandemic started, our Clinic of Neonatology approach was to separate the mother and infant at birth and require breastfeeding. This approach has changed due to a better organization of hospital resources and updated local guidelines according to international guidelines. We provide rooming-in and encourage breastfeeding for newborns delivered from mothers with a mild or asymptomatic form of COVID-19. If the diagnosis of infection in the newborn is confirmed, isolation from the mother is no longer necessary. Breastfeeding is encouraged in these cases if maternal and neonatal health allow it [30]. In the three cases presented, the newborns were moved together with the mothers after establishing the diagnosis of infection in neonates because the symptoms were minor or moderate. From that moment on, all newborns were exclusively breastfed.

Studies show that most newborns infected with SARS-CoV-2 remain asymptomatic or develop a moderate form of the disease [31,32]. In our case, two newborns had an unremarkable physical examination, and one patient presented mild respiratory distress syndrome, which may be linked to COVID-19 infection. The outcome was favorable, and improvements were observed after oxygen and antibiotic therapy. A case of neonatal distress syndrome in a preterm neonate delivered to an infected woman is described in the study of Zeng et al. (2020). Good progress was observed after non-invasive ventilation and medication [23].

Based on the literature, there is a hypothesized link between immune dysregulation caused by SARS-CoV-2 infection and oral manifestation in COVID-19-positive patients [33,34]. Candida infection in newborns is associated with increased morbidities and mortality [35]. Newborns diagnosed with COVID-19 have decreased immunity, representing significantly higher risk of acquiring fungal infections. The colonization of Candida albicans mainly causes oropharyngeal candidiasis [36]. The spread of Candida colonization in the body is a strong predictor of invasive candidiasis. By establishing an early diagnosis and instituting prompt infection treatment, the evolution of these cases is most often favorable [37].

By observing that all three newborns developed oral candidiasis, we can assign a higher risk of developing oral fungal lesions secondary to coronavirus infection. Buonsenso describes a late neonatal-onset SARS-CoV-2 infection after discharge with a negative test. They highlight the importance of a follow-up with newborns [38]. Monitoring cases of disease with persistent symptoms and the newborn’s development, highlighting possible sequelae that appear later in life are essential [31,39]. Therefore, the mothers included in this presentation received, at the discharge, the recommendation to consult the family doctor to record and monitor the subsequent evolution of the newborns [39].

Our study is limited by its small sample size and retrospective design and provides few SARS-CoV-2 positive newborns, although our Clinic of Neonatology is part of the largest tertiary maternity care unit in West Romania. In addition, there was no standardized protocol for blood sample tests for positive SARS-CoV-2 newborns. Therefore, there was a discrepancy between some laboratory determinations. Elaborating on a unanimously accepted protocol for clinical and paraclinical management of SARS-CoV-2 positive neonates could bring further clarifications or essential findings.

## 5. Conclusions

The epidemiological burden created by COVID-19 hangs heavily on every country. However, many aspects of COVID-19 still lack clarifying evidence, particularly for the population of newborn infants. Nevertheless, we believe these findings bring relevant clinical and paraclinical outcomes on COVID-19-positive newborns and the concern for vertical transmission of SARS-CoV-2. We conclude that vertical transmission is rare in late pregnancy. Nevertheless, numerous questions remain to be addressed concerning the effects of SARS-CoV-2 infection in neonates and require more research. In addition, more studies are needed for the standardized management of newborns exposed to or infected with SARS-CoV-2.

## Figures and Tables

**Table 1:** Characteristics of the newborns.

Parameters	Patient 1	Patient 2	Patient 3
**Demographic and anthropometric characteristics**			
Place of birth	CEHPBT	CEHPBT	Secondary maternity
Gestational age (weeks)	38	39	39
Type of birth	Vaginal	C-section	C-section
Presentation at birth	Cephalic	Cephalic	Cephalic
Gender	male	male	male
Weight at birth (g)	2990	3270	2900
Length at birth (cm)	50	52	50
Head circumference (cm)	33	34	31
Chest circumference (cm)	33	34	32
Ponderal index (^2^PI)	2.39	2.32	2.32
Classification based on PI	AGA	AGA	AGA
**Transition to extrauterine life—Apgar score**			
Appearance	General cyanosis	Acrocyanosis	Acrocyanosis
Pulse	>100	>100	>100
Grimace	PRS	PRS	PRS
Activity	Active	Active	Active
Respiration	Vigorous cry	Vigorous cry	Vigorous cry
At 1 min	8	9	9
At 5 min	10	10	10

C-section—cesarean section; PI—ponderal index was calculated fetal weights (in grams) × 100/fetal length (in centimeters); AGA—appropriate for gestational age; PRS = prompt response to stimulation.

**Table 2:** Clinical findings.

Parameters	Patient 1	Patient 2	Patient 3
**RDS**	mild	none	none
**Jaundice**			
DOL—1	moderate	none	none
DOL—2	moderate	mild	mild
DOL—3, 4	moderate	mild	moderate
DOL—5, 6	intense	mild	moderate
DOL—7	moderate	in regression	in regression
**Oral candidiasis**			
DOL—1, 2	no	no	no
DOL—3–5	yes	yes	yes
DOL—6	yes	no	yes
DOL—7	yes	discharged	discharged
**Nappy rash**			
DOL—1–3	no	no	no
DOL—4–7	yes	no	no

RDS—respiratory distress syndrome; jaundice—mild = total bilirubin <8.8 mg/dL; moderate = total bilirubin between 8.8–14.7 mg/dL; severe = total bilirubin > 14.7 mg/dL.

**Table 3:** Laboratory tests during hospitalization.

Parameters	Patient 1			Patient 2			Patient 3	Reference Range
	DOL-1	DOL-3	DOL-5	DOL-1	DOL-2	DOL-3	DOL-2	Age 0–month
Leukocytes	11.2	8.18	9.00	20.52	12.38	NA	12.76	9–30 × 10^3^/μL
Granulocytes	6.3	4.19	1.21	13.87	8.50	NA	7.78	1–20 × 10^3^/μL
Lymphocytes	4.3	1.94	5.47	5.11	2.60	NA	2.95	2–11 × 10^3^/μL
Hemoglobin	18.1	16.8	17.5	16.5	16.8	NA	19.2	13.4–19.9 g/dL
Hematocrit	51.9	46.1	48.6	47.9	47.7	NA	52.5	42–65%
Thrombocytes	253.000	172.000	178.000	183.000	180.000	NA	246.000	242.000–378.000/μL
^1^ CRP	7.4	0.99	<5	32.36	NA	10.35	<0.5	<0.5 mg/dL
^2^ ALT	33	NA	38	24	NA	NA	12	0–35 U/L
^3^ AST	86	NA	69	50	NA	NA	73	14–36 U/L
^4^ CK	NA	NA	NA	305	NA	NA	942	65–580 U/L
^5^ TB	6.2	11.2	13.6	-	NA	NA	8.55	<6.0 mg/dL
^6^ LDH	NA	NA	NA	668	NA	NA	735	160–450 U/L
Ferritin	NA	359	627	216	NA	NA	92	25–200 μg/L
IgG anti SpikeS1 quantitative	NA	NA	<2	<2	NA	NA	187	<17.8 ^7^ BAU = NEGATIVE;>17.8 BAU = POSITIVE
Anti-SARS-CoV-2 TOTAL (IgA IgM IgG)	NA	NA	0.05	0.09	NA	NA	NA	0.00–0.99 ^8^ s/co = UNEREACTIVE;≥1.00 REACTIVE

^1^ CRP = C-reactive protein; ^2^ ALT = alanine aminotransferase; ^3^ AST = aspartate aminotransferase; ^4^ CK = creatin phosphokinase; ^5^ TB = total bilirubin; ^6^ LDH = lactate dehydrogenase; ^7^ BAU = binding antibody units; ^8^ s/co = signal/cut-off.

## Data Availability

The data presented in this case series are available on request from the corresponding author.
